# Water Stress and Seed Color Interacting to Impact Seed and Oil Yield, Protein, Mucilage, and Secoisolariciresinol Diglucoside Content in Cultivated Flax (*Linum usitatissimum* L.)

**DOI:** 10.3390/plants12081632

**Published:** 2023-04-12

**Authors:** Sara Zare, Aghafakhr Mirlohi, Mohammad R. Sabzalian, Ghodratollah Saeidi, Mehmet Zeki Koçak, Christophe Hano

**Affiliations:** 1 Department of Agronomy and Plant Breeding, College of Agriculture, Isfahan University of Technology, Isfahan 84156 83111, Iran; 2 Department of Herbal and Animal Production, Vocational School of Technical Sciences, Igdir University, 76000 Igdir, Turkey; 3 Department of Chemical Biology, Eure & Loir Campus, University of Orleans, 28000 Chartres, France

**Keywords:** flaxseed, lignans, unsaturated fatty acids, secondary metabolites, heat mapping

## Abstract

Flaxseed (*Linum usitatissimum* L.) is a plant with a wide range of medicinal, health, nutritional, and industrial uses. This study assessed the genetic potential of yellow and brown seeds in thirty F4 families under different water conditions concerning seed yield, oil, protein, fiber, mucilage, and lignans content. Water stress negatively affected seed and oil yield, while it positively affected mucilage, protein, lignans, and fiber content. The total mean comparison showed that under normal moisture conditions, seed yield (209.87 g/m^2^) and most quality traits, including oil (30.97%), secoisolariciresinol diglucoside (13.89 mg/g), amino acids such as arginine (1.17%) and histidine (1.95%), and mucilage (9.57 g/100 g) were higher in yellow-seeded genotypes than the brown ones ((188.78 g/m^2^), (30.10%), (11.66 mg/g), (0.62%), (1.87%), and (9.35 g/100 g), respectively). Under water stress conditions, brown-seeded genotypes had a higher amount of fiber (16.74%), seed yield (140.04 g/m^2^), protein (239.02 mg. g^−1^), methionine (5.04%), and secondary metabolites such as secoisolariciresinol diglucoside (17.09 mg/g), while their amounts in families with yellow seeds were 14.79%, 117.33 g/m^2^, 217.12 mg. g^−1^, 4.34%, and 13.98 mg/g, respectively. Based on the intended food goals, different seed color genotypes may be appropriate for cultivation under different moisture environments.

## 1. Introduction

Flaxseed (*Linum usitatissimum* L.) is an excellent source of lignans, mucilage, and oil (30 to 44%). The oil is rich in polyunsaturated fatty acids, particularly alpha-linolenic acid (omega-3) and linoleic acid (omega-6) [1]. The oil of flaxseed is still fundamentally used as a source of industrial oil due to its high alpha-linolenic acid (ALA) content (up to 50% of total fatty acids); also, it is rich in polyunsaturated fatty acids (73%), monosaturated fatty acids (18%), and low in saturated fatty acids (9%) [1,2]. The fatty acids of flaxseed oil are mainly composed of ALA, linoleic acid (LA), oleic acid, stearic acid, and palmitic acid, making it a very beneficial daily diet for human health [3,4,5]. Flaxseed is high in protein and soluble and insoluble dietary fibers, which are extremely useful to human health [6]. The amount of lignans is different in plants, but the highest amount has been reported in flaxseed and is usually found as glycosylated in oligomeric chains [7]. The main pharmacologically active lignans in flaxseed are secoisolariciresinol diglucoside (SDG) and secoisolariciresinol (SECO) [8]. SECO can be glycosylated via the action of UDP-glucosyltransferase (UGT) to yield over 95% of the total lignan components in flaxseed [9]. Human gut bacteria further metabolize flax lignans to mammalian lignans (enterodiol and enterolactone), which have been shown to significantly reduce the risk of cancer, especially in the breast, colon, and prostate, and alleviate menopausal symptoms [10,11,12].

Flaxseed is one of the essential sources of polysaccharides (about 28% of seed weight), in both water-soluble and insoluble forms in an approximate ratio of 20:80 to 40:60. The main fractions of insoluble polysaccharides are cellulose and lignin and the water-soluble form is mucilage [13]. Mucilage with more than 8% of seed mass is found in the epidermal cell layer of the flax seed coat. The major component of mucilage is pectin, and the raw mucilage of the flaxseed coat can be divided into two groups: neutral (arabinoxylan) and acidic (pectin-like) fractions [14,15]. The effects of fiber and mucilage in flaxseed on the gastrointestinal tract of humans and animals have been studied and shown to cause more and easier, mild purgative, increased satiety (while insulin levels decreased), and decreased fasting cholesterol and LDL levels [16,17].

Flaxseed is recognized by variety or seed color (brown and yellow). Seed color in flaxseed can have an association with other valuable features. Most of flaxseed accessions are brown-seeded (high in ALA); however, yellow-seeded ones are also found [1]. Considering the increasing demand for flaxseed, developing new varieties with the best combination of desired characteristics is needed. High seed yield, higher and more stable nutraceutical composition, balanced and stable fatty acid profile, and more suitable products for the food market are among the most crucial breeding goals [18,19]. In this respect, it is very important and necessary to know the interaction effect of seed coat color (yellow and brown) with different environmental conditions, considering the trend of changing environmental conditions in the world. Carefully designed hybridization between contrasting parents usually creates the desired progeny populations for such studies.

There is very little information and fewer studies on the response of segregating brown and yellow-seeded flax genotypes’ responses to water stress concerning the amount of lignans, fatty acid compositions, and diversity of mucilage [20]. In this study, a group of selected brown and yellow F4 progenies derived from hybridization between contrasting parents regarding these seed colors were field evaluated under different irrigation regimes (control 50% and drought stress 80% of soil available water). The objectives were to study the effects of genotype, seed color, and water stress on seed yield, seed oil, fatty acids, fiber and its components, lignans, mucilage, protein, and amino acids contents. These results might promote the production of flaxseed under different moisture environments for use in the cosmetic, pharmaceutical, and food industries.

## 2. Results

### 2.1. Analysis of Variance (ANOVA) for Different Biochemical Characteristics of Flaxseed

The results obtained from the analysis of the variance of data for different traits over two moisture environments (normal and water stress) are reported in Table 1 and Table 2. Water stress affected all measured traits significantly (*p* < 0.0), except for aspartic acid (AA), α- Linolenic acid (ALA), and acid detergent fibers (ADF) and the effect of entries (families and parents) was significant for all traits. The effect of families for all traits except AA and moisture (Moi), the effect of yellow seed color within the families for all traits except hemicellulose (HCEL) and fiber (FIB), and the effect of brown seed color within the families were significant for all traits. The effect of yellow versus brown genotypes within families was not significant for lignin (ADL), cellulose (CEL), HCEL, palmitic acid (PA), AA, protein (PRO), Moi, and arginine (Arg) (Table 1 and Table 2). The effect of parents and their yellow and brown genotypes were not significant for seed yield (SY), FIB, Moi, and ash content, except in the yellow families, ash was significant at the 5% level, and the effect of brown genotypes within parents was not significant for AA and Met (methionine). The difference of yellow versus brown seeded within parents was significant only for SDG, ALA, AA, ADF, and Arg. Additionally, the effect of family versus parents was significant for all traits except ADF, ALA, PA, AA, PRO, Met, and ash content (Table 1 and Table 2).

Environment and entries interactions were not significant for oil content, Muc, and Moi traits. The environment and yellow and brown within crosses interactions were significant for most traits except oil in both seed coat color and ADF, OA, and AA content in brown-seed families and FIB in yellow-seed families. The interactions of environment and yellow and brown families within the parents were significant in most traits except oil content. Additionally, the interaction of environment and brown-seeded families within the parents for ADF, FIB, and Moi and in yellow-seeded families for SY, ALA, and His were not significant (Table 1 and Table 2).

The means of studied traits for the parents and their derived families under two water conditions of non-stress (normal) and water stress are presented in Table 3, Table 4 and Table 5.

#### 2.1.1. Seed Yield and Seed Oil Characteristics

**Figure 1:**
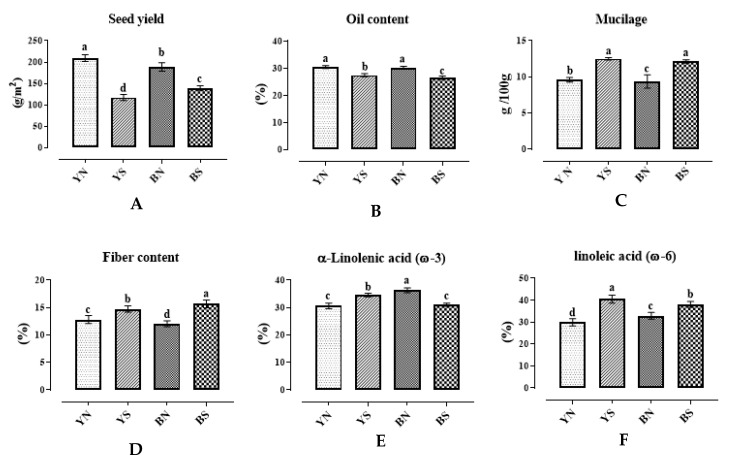
Diagrams of seed yield (**A**), oil content (**B**), mucilage (**C**), fiber content (**D**), α- Linolenic acid (omega-3) (**E**), and linoleic acid (omega-6) (**F**) extracted from flax families under water stress and normal irrigation conditions. A same letter above the columns for each trait (diagram) indicates that the values are not statistically different (*p* > 0.05). Values are mean ± standard error. Letters y, b, n, and s denote yellow color, brown color, normal conditions, and water stress conditions, respectively.

When comparing the seed yield means under normal and water stress conditions (Table 3), a considerable variation among genotypes was observed. Results showed a reduction in seed yield under water stress conditions in both families with brown and yellow seeds, and this decrease was seen more in yellow than brown seeds (Figure 1A). The highest seed yield under normal water conditions was observed in family 83y (271.1 g/m^2^) and the lowest in family 26y (130.1 g/m^2^). The highest seed yield under water stress was observed in family 61b (197.7 g/m^2^) and the lowest in family 26y (34.23 g/m^2^) (Table 3).

Water stress influenced seed oil content with a significant reduction on the average of all genotypes but with a considerable variation among them. Oil content was higher in families with yellow seeds than the brown-seeded ones under normal and water stress conditions, but there was no significant difference in the amount of oil between families with yellow and brown seed coat color under normal and water stress conditions (Figure 1B).

Under normal water conditions, the highest oil content was observed in parent B1 (41.7%) and the progenies of family 71y (39.92%). The lowest oil content was marked in parent B2 (25.7%) and the progenies of family 28b (26.46%). Under water stress conditions, the highest oil content was observed in parent Y6 (34.9%) and the progenies of family 48b (33.71%). The lowest oil content was observed in the family 26y (25.1%) (Table 3). Comparing the mean fatty acid profiles showed that families with the highest values of OA, LA, ALA, and PA under normal moisture conditions belonged to 43b (20.6%), 43b (49.09%), 56b (44.425%), and 83y (9.6%), respectively. However, the lowest values were noted in parents B4 (8.2%), Y7 (20.7%), 83y (22.32%), and 81y (3.7%), respectively (Table 4). Under water stress conditions, the amount of some fatty acids such as OA and LA in most families increased, and the highest amount of OA was observed for family 47y (22.7%), and the highest LA was observed for parent Y5 (55.3%) and in family 43b (54.28%). The lowest amount of OA was noted in family 26y (9.4%), and lowest LA was observed for parent Y6 (28.1%) and the progenies of family 56b (31.48%). Under water stress conditions, the highest amounts of ALA and PA were observed in the family 17y (37.6%) and 17y (9.01%), and the lowest levels were observed in 13b (25.2%) and 48b (3.02%), respectively (Table 4).

The omega-6 to omega-3 ratio (Rate(ω-6): (ω-3)), in most cases, increased under water stress conditions in both families with yellow and brown seeds, but the amount of this increase was higher in yellow-seeded families than brown-seeded ones (Table 5). The highest ratio of linoleic acid (omega-6) to ALA (omega-3) ((ω-6)/(ω-3)) was observed in the parent B4 (1.8/1) and progenies in the family 43b (1.6/1) and the lowest one was seen in the family 72y (0.6/1) at normal moisture conditions. At the same time, the highest ratio of ((ω-6)/(ω-3)) was observed in family 75y (1.9/1) and the lowest value of these traits was marked in parent Y8 (0.9/1) under water stress (Table 4).

Additionally, the comparison chart of LA (omega-6) and ALA (omega-3) levels showed that the highest amount of LA and ALA under normal moisture conditions was observed in families with brown seeds. In contrast, in water stress conditions, families with yellow seeds had higher levels of LA and ALA (Figure 1E,F). Given that environmental stresses cause a decrease in traits, the seed oil components were affected due to water stress, and it was more considerable in yellow seeds than the brown ones. The amount of ALA decreased in brown seeds under water stress, while it increased in yellow ones. At the same time, the amount of LA increased under moisture stress for both seed colors, though to a greater extent in yellow-seed families. The results showed that the oil quality in yellow seeds was higher than in brown ones under both moisture treatments.

#### 2.1.2. Protein and Amino Acids Contents

Comparing the average amount of protein and amino acids in flaxseed under water stress and normal conditions showed that water stress positively influenced protein and amino acid content differently in brown- and yellow-seeded families. The increase was more in families with brown seed color than yellow-seeded families, with some exceptions. Comparison of the means for crude protein and amino acid contents showed that the highest amounts of protein, methionine (Met), histidine (His), aspartic acid (AA), and arginine (Arg) under normal moisture conditions belonged to the families 47y (272.1 mg. g^−1^), 12b (3.22%), 43b (3.57%), Y6 and Y7 (2.95%), and 82y (2.56%), respectively (Table 5). Under this condition, the lowest values of these traits were observed in the families B3 (104.9 mg. g^−1^), 72y (1.09%), B3 (0.91%), 23b (1.23%), and B2 (0.28%). Under water stress conditions, the highest amount of crude protein belonged to the parent Y5 (307.5 mg. g^−1^) and progenies of the family 75y (293.46 mg. g^−1^), and the lowest belonged to the family 53b (153.7 mg. g^−1^). Additionally, under this condition, the highest amount of Met, His, AA, and Arg were observed in families 13b (6.45%), 83y (4.54%), B1 (5.01%), and 17y (1.85%), while the lowest values belonged to families 48b (2.96%), 23b (0.76%), 82y (0.89%), and 28b (0.21%), respectively (Table 5).

**Figure 2:**
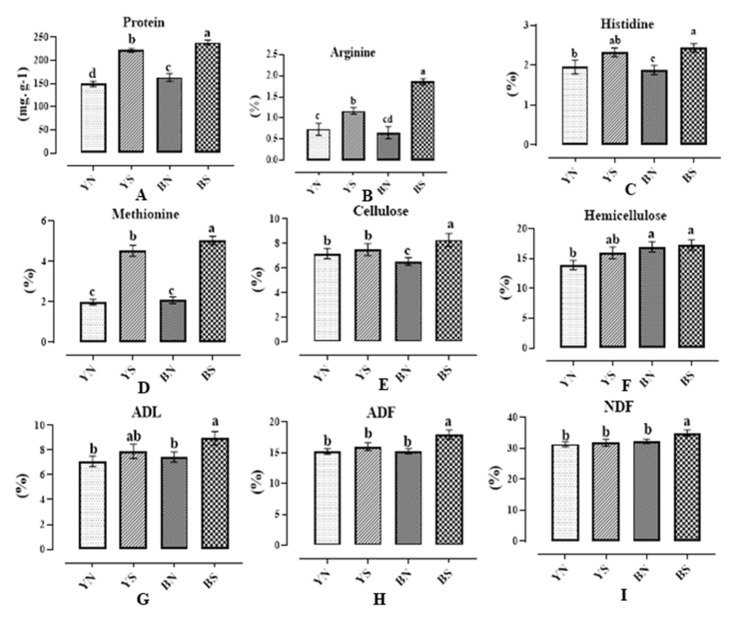
Diagrams of protein (**A**), arginine (**B**), histidine (**C**), methionine (**D**), cellulose (**E**), hemicellulose (**F**), lignin (**G**), acid detergent fibers (**H**), and neutral detergent fibers (**I**) extracted from flax families under water stress and normal irrigation conditions. A same letter above the columns for each trait (diagram) indicates that the values are not statistically different (*p* > 0.05). Values are mean ± standard error. Letters Y, B, N, and S denote yellow color, brown color, normal conditions, and water stress conditions, respectively.

The compared results of protein content (Figure 2A) showed that in both normal and water stress conditions, the amount of protein was higher in brown families. Additionally, water stress increased the amount of protein, which was much higher in brown families. In addition, amino acids increased under the influence of moisture stress, although there was no significant difference between yellow- and brown-seeded families under normal water conditions. Under water stress, brown-seeded families had the highest amount of arginine and methionine (Figure 2B, D). Regarding histidine, the result showed that it was increased under water stress conditions especially in families with brown seeds and there was significant difference between yellow and brown seed coat color under normal and water stress conditions (Figure 2C).

#### 2.1.3. Mucilage and Fiber Content of Seeds

The comparison of the means showed that water stress increased the amount of mucilage and fiber in the studied flax families. In most cases, the increase in the amount of total fiber and mucilage under stress conditions was higher in families with brown seed coat color than yellow. Under normal water conditions, the lowest and the highest amount of mucilage belonged to families 53b (6.06 g/100 g) and 26y (12.7 g/100 g), respectively. Additionally, the highest amount of mucilage under water stress conditions was observed in families 26y and 56b (13.7 g/100 g), and the lowest value was seen in the family 47b (8.75 g/100 g). The amount of mucilage in normal water conditions was more in yellow-seeded families than in brown seeded, while in the conditions of water stress, their average value was almost the same, so it is likely that the production of mucilage in brown-seeded families was more influenced by environmental conditions. The mucilage in flax seeds increased due to water stress in both families with yellow and brown seeds. Under water stress conditions, the average amounts of mucilage in yellow- and brown-seeded families were 12.49 and 12.13 g/100 g seed, respectively, while they were 9.59 and 9.35 g/100 g seed in normal water conditions (Figure 1C).

The total seed fiber content in yellow-seeded families was higher than brown ones at normal water conditions, and the amount of total fiber increased due to water stress. The increase was much higher in families with brown seed color (Figure 1D and Table 4) than the yellow ones. The results showed a three-fold increase in fiber content in some families with yellow seeds, such as the parent Y5. In contrast, in some other families, such as 17y, a decrease of 7% was observed. Still, in most families with yellow seed coat color, there was an increase of about 25% in the amount of seed fiber, while in most families with brown seed, a more than 40% increase in fiber content was observed in water stress conditions. Under normal water conditions, the highest amount of seed fiber was seen in the family 12b and the lowest in the family 47y. Under water stress conditions, the highest fiber content was observed for the parent Y5 and the family 23b, and the lowest amount was observed in the family 61b at this condition. Additionally, the highest levels of NDF, ADF, and ADL in normal water conditions belonged to genotypes 43b, 12b, and 32b, respectively. However, their lowest values were observed in 13b, 28b, and 72y. In water stress conditions, the highest amount of NDF was found in parent Y6, and the lowest value belonged to the family 83y. For ADF, the highest value was found in the parent Y7 and progenies of 34b, and the lowest value was observed in 28b. Additionally, the highest amount of lignin (ADL) in stress conditions was in family 34b and the lowest in family 28b (Table 3). The results of CEL and HCEL in Table 4 showed that the higher values of CEL and HCEL in normal water conditions were observed in families 72y, parent B4, and progenies of 61b, respectively. The lowest values belonged to the families 32b and 13b, respectively. In contrast, in water stress conditions, the highest values of CEL were found in parent Y7 and progenies of 12b, and the lowest one was observed for family 75y. The highest HCEL was seen in parent Y6 and progeny of 23b, and the lowest was in parent Y5 and progeny of 75y (Table 3).

In the diagram comparing the average of families with yellow and brown seeds under normal moisture conditions, no significant difference was observed between the values of cellulose, ADF, NDF, ADL, and hemicellulose, except hemicellulose in yellow-seeded families, while these traits increased under the influence of water stress, and their values in families with brown seeds were more than the families with yellow seeds (Figure 2E–I).

#### 2.1.4. Seed Lignans Content

**Figure 3:**
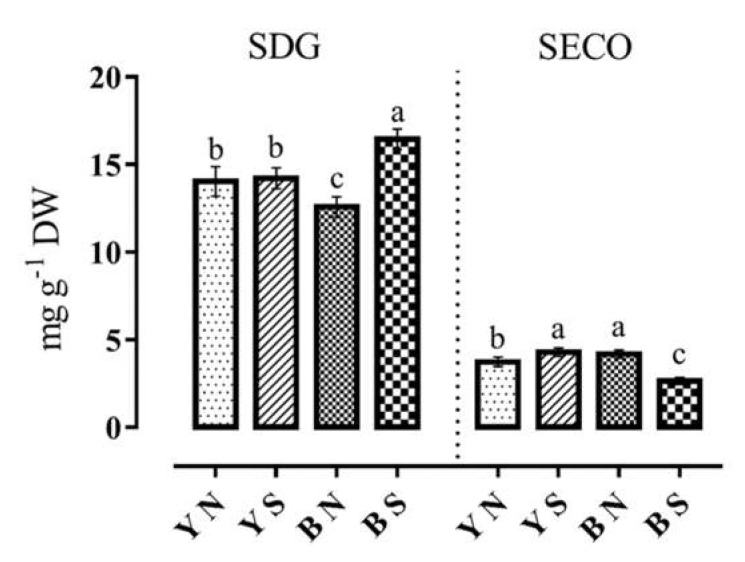
Diagrams of SDG content (secoisolariciresinol diglucoside, in mg g^−1^ dry weight) and SECO content (Secoisolariciresinol, in mg g^−1^ dry weight) extracted from families of flax under water stress and normal irrigation conditions. A same letter above columns for each trait (diagram) indicates that values are not statistically different (*p* > 0.05). Values are mean ± standard error. Letters Y, B, N, and S denote yellow color, brown color, normal conditions, and water stress conditions, respectively.

Comparing the mean of lignans showed that due to water stress, the amount of SDG increased by 35% in families with brown seed coat color and by 5.19% in families with yellow seed on average (Figure 3). However, in families 83y, 72y, 45y, and 47y, there was a 16 percent reduction in SDG. In some families, such as 47y and 47b, under normal water conditions, SDG was higher in yellow families, while under water stress conditions, the brown-seeded ones showed a 41% increase in SDG. The SDG mean comparison showed that the highest SDG values belonged to parent Y6 (22.21 mg. g^−1^) and progenies in the family 56b (21.56 mg. g^−1^) under water stress conditions and the family 83y (19.5 mg. g^−1^) in normal conditions. The lowest SDG values were observed in families 13b (9.5 mg. g^−1^) and 71y (6.37 mg. g^−1^) in water stress and normal conditions, respectively (Table 5). On the other hand, the highest amount of SECO in water stress conditions was observed in the family 83y (5.73 mg. g^−1^), and the lowest amount was found in family 48b (1.03 mg. g^−1^). In normal water conditions, the highest value of SECO was seen in family 47b (5.63 mg. g^−1^), while the lowest was observed in family 26y (1.67 mg. g^−1^) (Table 5).

Figure 3 shows the values of SDG and SECO under normal and water stress conditions in the two seed color categories of yellow and brown. The comparison of SDG in yellow-seeded families under normal and water stress conditions showed that SDG levels were almost stable and were not affected by moisture stress. In contrast, values in brown-seeded families showed that SDG increased significantly due to water stress, and the highest amount of SDG was observed in brown seeds in this condition (16.45 mg. g^−1^).

The SECO is a precursor converted to SDG in a process with an unknown path. A comparison of SECO values showed that its highest amount was observed in brown-seeded families in normal moisture conditions. Under water stress conditions, the highest amount of SECO was detected in yellow-seeded families, while the lowest amount of SECO was also observed in brown-seeded families (Figure 3).

### 2.2. Principal Component Analysis (PCA) and Heat Mapping

**Figure 4:**
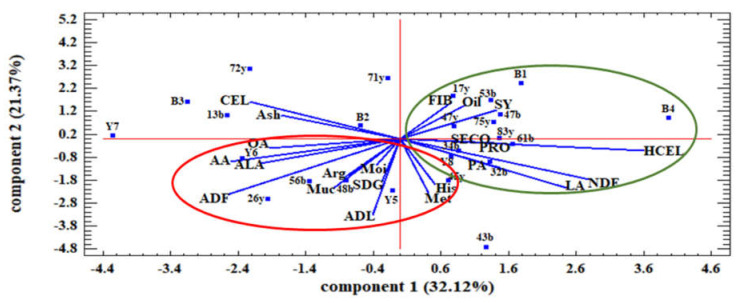
Principal component analysis of the relationship among the studied traits and the distribution of families of flax relative to these traits under normal water conditions. SY: seed yield. Oil: oil content. NDF: neutral detergent fibers. ADF: acid detergent fibers. ADL: lignin. CEL: cellulose. HCEL: hemicellulose. Muc: mucilage. PRO: protein content. FIB: fiber content. OA: oleic acid. LA: linoleic acid. PA: palmitic acid. ALA: α- Linolenic acid. Met: Methionine. His: histidine. Arg: arginine. AA: aspartic acid. Moi: moisture. SDG: secoisolariciresinol diglucoside. SECO: secoisolariciresinol.

**Figure 5:**
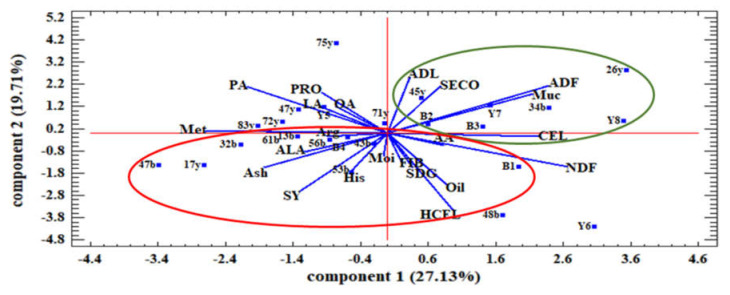
Principal component analysis of the relationship among the studied traits and the distribution of flax families relative to these traits under water stress conditions. SY: seed yield. Oil: oil content. NDF: neutral detergent fibers. ADF: acid detergent fibers. ADL: lignin. CEL: cellulose. HCEL: hemicellulose. Muc: mucilage. PRO: protein content. FIB: fiber content. OA: oleic acid. LA: linoleic acid. PA: palmitic acid. ALA: α- Linolenic acid. Met: methionine. His: histidine. Arg: arginine. AA: aspartic acid. Moi: moisture. SDG: secoisolariciresinol diglucoside. SECO: secoisolariciresinol.

The results of the principal component analysis of the studied families and traits under normal and water stress conditions are shown in Figure 4 and Figure 5, respectively.

Under normal moisture conditions, the principal component analysis showed that the first two components explained 53.49 percent of the total variance. The first principal component (PC1) had a positive and significant correlation with seed yield, oil content, FIB, PRO, HCEL, LA, PA, NDF, and SECO and a negative correlation with ash, OA, and CEL. In contrast, the second principal component (PC2) negatively correlated with Muc, SDG, ADL, His, Met, ADF, ALA, and AA (Figure 4).

Additionally, the results showed that most traits in the first component had a negative correlation with the traits in the second component. Based on genotype discrimination in the two areas of the biplot diagram, it was clear that the families in the fourth quarter had the highest values for SY, oil content, FIB, PRO, and SECO. In this respect, families 53b, 17y, 47b, 34b, 61b, 47y, 83y, B1, and B8 were considered superior families. In contrast, families in the first quarter had high values of mucilage and SDG, such as 56b, 26y, 48b, Y5, and Y6.

The results of principal component analysis under water stress conditions showed that the first two components explained more than 46.84% of the variance (Figure 5). The first principal component (PC1) had a positive and high correlation with Muc, ADF, CEL, and NDF and negatively correlated with Met, LA, and Arg. The second principal component (PC2) had a positive high correlation with ADL, SECO, and PRO and negatively correlated with SDG, FIB, HCEL, SY, and oil. The positive value of the first component symbolized high mucilage and fiber, and the negative value of the second component symbolized a lower value of SY, oil, and SDG. Therefore, the families in the fourth quarter circle, including 26y, 34b, 45y, B3, Y8, and B2, had the highest amount of mucilage, SECO, and CEL and the lowest amount of SY, oil, and SDG. In contrast, the families that were in the circle of the first quarter, including 17y, 53b, 32b, 43b, 48b, 47b, and 56b, had the highest SY, oil, FIB, and SDG and the lowest amount of SECO and mucilage under water stress conditions (Figure 5).

**Figure 6:**
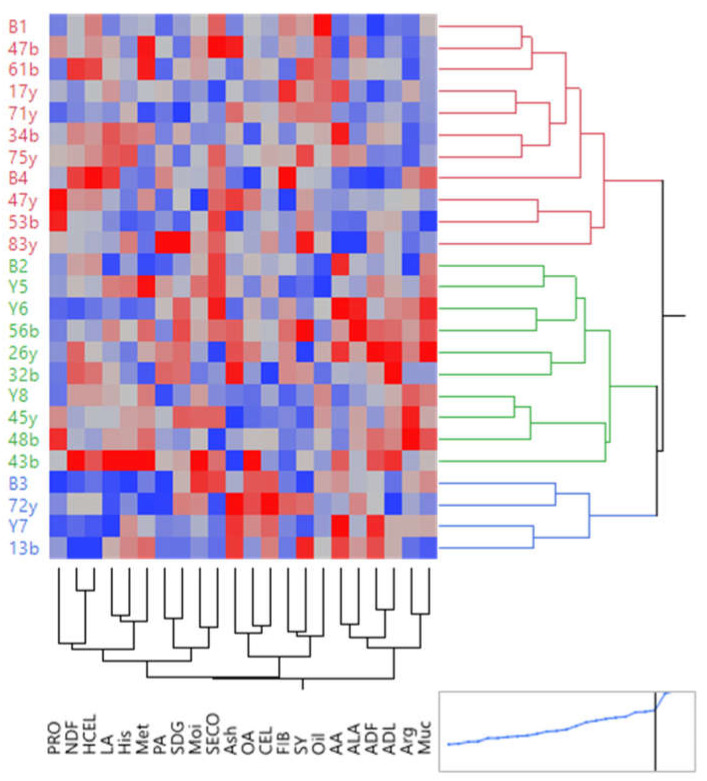
Heat-mapping diagram related to the studied traits of flax families under normal conditions. The dark blue color indicates the lowest value of the characters, and the dark red color indicates the highest value. SY: seed yield. Oil: oil content. NDF: neutral detergent fibers. ADF: acid detergent fibers. ADL: lignin. CEL: cellulose. HCEL: hemicellulose. Muc: mucilage. PRO: protein content. FIB: fiber content. OA: oleic acid. LA: linoleic acid. PA: palmitic acid. ALA: α- Linolenic acid. Met: methionine. His: histidine. Arg: arginine. AA: aspartic acid. Moi: moisture. SDG: secoisolariciresinol diglucoside. SECO: secoisolariciresinol.

For grouping the families based on the studied traits, heat mapping was used and graphically showed the status of each family concerning all the attributes. In this graph, the dark blue color shows the lowest value of a trait, and the dark red indicates the highest (Figure 6). In this respect, the families fell into three groups (A, B, and C) under normal water conditions. Group A included parents and families B1, 47b, 61b, 17y, 71y, 34b, 75y, B4, 47y, 53b, and 83y, with the highest values of seed yield, oil, and fiber content and the lowest values of amino acids, Muc, and LA. Additionally, in group A, the family 83y had the highest seed yield and SDG under normal moisture conditions. Group B included parents and families B2, Y5, Y6, 56b, 26y, 32b, Y8, 45y, 48b, and 43b, with high levels of amino acids, mucilage, ADF, ADL, and SECO and low levels of seed yield, oil content, CEL, and fiber content. In addition, family 43b in this group had the highest values for most of the studied traits, except for oil and seed yield. Group C included four families, B3, 72y, Y7, and 13b, with the minimum values for most traits, except for AA, ADF, CEL, OA, and ALA (Figure 6).

**Figure 7:**
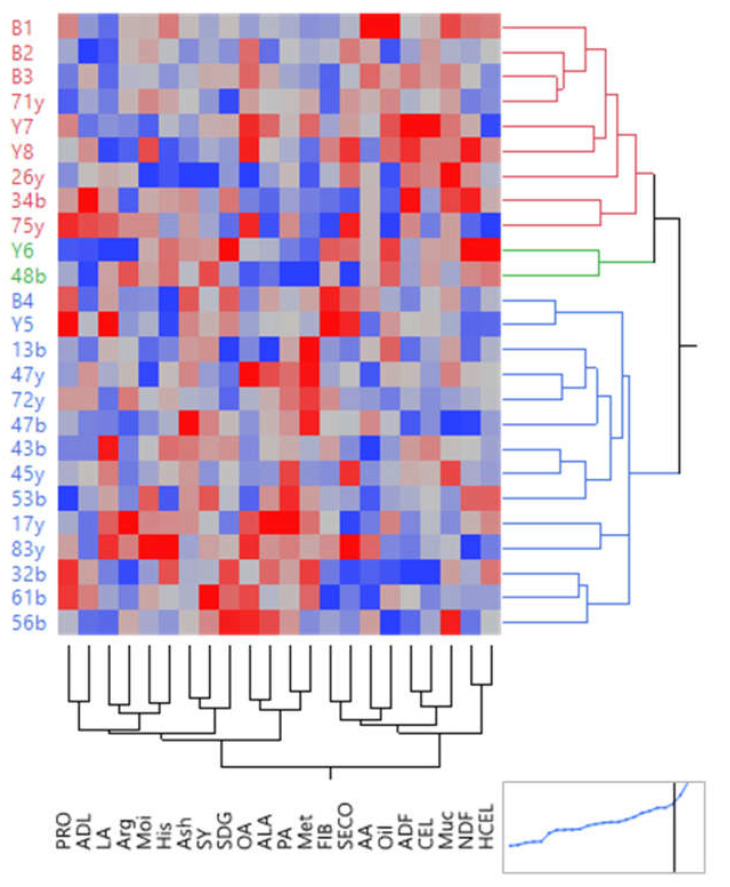
Heat mapping diagram related to the studied traits of flax families under water stress conditions. The dark blue color indicates the lowest value of the characters, and the dark red color indicates the highest value. SY: seed yield. Oil: oil content. NDF: neutral detergent fibers. ADF: acid detergent fibers. ADL: lignin. CEL: cellulose. HCEL: hemicellulose. Muc: mucilage. PRO: protein content. FIB: fiber content. OA: oleic acid. LA: linoleic acid. PA: palmitic acid. ALA: α- Linolenic acid. Met: methionine. His: histidine. Arg: arginine. AA: aspartic acid. Moi: moisture. SDG: secoisolariciresinol diglucoside. SECO: secoisolariciresinol.

Similar to the normal moisture conditions, the results of heat mapping for water stress conditions divided the studied entries into three groups (A, B, and C). Group A included parents and families B1, B2, B3, Y7, Y8, 71y, 26y, 34b, and 75y with the highest oil, CEL, SECO, ADF, FIB, and mucilage. This group was weak regarding SDG, ALA, seed yield, and amino acids (Figure 7). Group B consisted of two entries of parent Y6 and progeny of 48b. Parent Y6 had the highest SDG, NDF, and HCEL values, while family 48b was average for most traits. Group C included parents and families B4, Y5, 13b, 47y, 72y, 47b, 43b, 45y, 53b, 17y, 83y, 32b, 61b, and 56b with high amounts of Met, PA, ALA, OA, Ash, SY, and SDG and the lowest quantity of oil, CEL, Muc, HCEL, and SECO (Figure 7).

## 3. Discussion

### 3.1. Association of Brown Seed Color with the Amount of Protein, Fiber, and Lignan (SDG) Content of Seeds in Conditions of Water Stress

The initial objective of this study was to investigate the effect of water stress conditions and seed coat color on seed yield and quality traits. In general, water stress causes a decrease in seed yield, associated with reduced assimilate synthesis required for seed filling [21]. Flax families and parents with brown seed coats had higher seed yield stability than yellow seed ones under water stress conditions, probably due to a higher amount of secondary metabolites such as lignans and tannins [22]. This association has been found in other crops, such as sesame, where seed yield reduction due to water stress was significantly lower in darker seed coat color genotypes than in lighter ones. At the same time, the secondary metabolites such as caffeic, ferulic, ellagic acids, and tannins were higher in darker seeds [23].

Flaxseed is rich in protein and amino acids such as arginine, aspartic acid, histidine, and methionine. Due to their antioxidant properties, they reduce the risk of cancer and effectively lower plasma cholesterol and triglycerides [24]. Water stress positively affected the amount of protein and amino acids. In the present study, in most families with brown seeds, water stress increased the amount of protein and amino acids more than the yellow-seeded ones. Foroud et al. [25] and Tavares et al. [21] also observed an increasing amount and quality of protein under water deficit conditions in other oil seed crops such as soybean. When plants are stressed, they usually accumulate protein and amino acids. Furthermore, the accumulation of amino acids can have various roles in plants, ranging from osmolyte function, ion transport, modulation of the stomatal opening, and the detoxifying of heavy metals. They also influence how enzymes are synthesized and remain active in redox homeostasis and gene expression that, as a result, causes sustainability and resistance in the plant under biotic and abiotic stresses [26].

Seed oil content decreased under water stress in brown-seeded families. Previous studies found a negative correlation between oil and protein content in soybean seeds under water stress conditions that can also be explained by the competition of synthesis pathways by carbon skeletons, changes in accumulation, and the distribution of nutrients [21]. Similarly, in this study, the amount of fiber and fiber components such as ADF, NDF, CEL, HCEL, and lignin in seeds increased due to water stress, but it was more in brown-seeded genotypes. Many factors affect the amount of fiber in seed, including environmental conditions (water, temperature), the amount of nutrients available to the plant, and the phase of plant development. Due to moisture stress, an increased amount of cellulose and hemicellulose has been reported in plants, suggesting that their production is most affected by water restriction, which makes the plant more resistant to moisture stress [13]. As the brown-seeded families were more tolerant to water stress than yellow seed ones, it can be concluded that fiber content may have played an essential role in flax drought tolerance.

Flax lignans also impart health benefits, particularly in controlling cancers, due to their strong phytoestrogenic and antioxidant properties [27]. Studies have shown that lignans are mainly localized in the secondary wall of the sclerite cell layer of the outer integument of the seed [28,29]. Like many other polyphenolic compounds in plants, lignans, due to their antioxidant, antiviral, antibacterial, and antifungal properties, play a critical role in plant defense mechanisms [27,30,31]. Plant lignan content is affected by various factors such as genotype, tissue type, geographical origin, environmental conditions, nutrition, and plant maturity. Indeed, SDG and SECO are lignans and secondary metabolites that can cause plant resistance to adverse environmental conditions. Our results showed that the amount of lignans increased under water stress.

Under normal water conditions, the amount of SDG was higher in yellow-seeded families than the brown counterparts and stayed the same under water stress conditions. In contrast, water stress significantly increased SDG in brown-seeded families with higher values than yellow ones in this condition. Therefore, it seems that more SECO is converted to SDG under water stress conditions in brown-seeded genotypes. These findings were consistent with the results of Shulha et al. [32] and Ražná et al. [27], who showed that the amount of lignans in the plants increased under stressful conditions. This can increase the plant’s resistance to adverse environmental conditions and stresses. Induction of water stress through appropriate irrigation may be suggested for higher levels of lignans in brown seed flax, considering their antioxidant and anticancer properties.

### 3.2. Effect of Water Stress on the Quality of Oil, Mucilage, Lignan (SECO), and Seed, and Oil Yields in Yellow Seeds

As illustrated in Figure 1B, water stress negatively affected seed oil content in both colored seed coat genotypes of flax. Although the difference between the yellow and brown families was not significant, this effect was more considerable in brown-seeded families than the yellow ones. This result is consistent with Yeloojeh et al. [33]. Additionally, in this study, seed yield decreased in water stress conditions in yellow-seeded families, showing a positive correlation between oil yield and seed yield under water stress conditions. These findings were also observed in the study of Kermani et al. [23] in sesame, Kiprovski et al. [34] in soybean, and Mittapalli et al. [35] in flaxseed. The downregulation of enzymes involved in fatty acids biosynthesis (such as acetyl-CoA carboxylase, 1,3-ketoacyl-CoA synthase) may lead to a lower oil content under water stress conditions [36].

Contrary to oil yield, the amount of oil fatty acids such as OA and LA increased in most families under water stress conditions, especially families with yellow seeds. In contrast, the amount of ALA and PA decreased. In general, under water stress conditions, the amount of all unsaturated fatty acids in yellow-seeded families was higher than the brown-seeded ones, reflecting the higher nutritional value of the oil. Another study has also shown that the amount of unsaturated fatty acids, including oleic acid, LA, and ALA is strongly influenced by environmental conditions [37]. Omega-3 and omega-6 unsaturated fatty acids are not synthesized in the human body, and their presence is essential in the diet. Therefore, the higher the ALA content, the higher the nutritional value of the seed [38]. Water stress increased some unsaturated fatty acids and the ratio of ω-6 to ω-3, with the values being higher in yellow-seed families, suggesting a better oil quality of yellow-seeded flax under water stress conditions.

Under abiotic stresses such as water deficiency, plants try to survive by increasing the production of substances such as mucilage and fiber to keep more water. Mucilage is a polysaccharide mixture with highly variable chemical constituents which plays a vital role in drought tolerance by modulating water retention and ion homeostasis in plants [39]. It has been suggested that the ability of mucilage to hydrate may offer a mechanism for drought tolerance to plants [40]. In addition, the natural polysaccharides found in plant mucilage have excellent antioxidant activity, which prevents cell damage caused by reactive oxygen species.

From the industrial and food perspective, mucilage has many applications, including pharmaceuticals, cosmetics, textiles, paper, and paint production [41]. In the present study, in both moisture conditions, the amount of mucilage in yellow-seeded families was slightly more than the brown ones. Similarly, the study of Diederichsen et al. [42] showed that the amount of mucilage in light-colored seeds of flax, especially yellow ones, is more than the dark-colored seeds.

Lignans are essential in plant defense against biotic and abiotic stresses [43]. Contrary to SDG, a comparison of genotypes for SECO values showed that brown-seeded families contained higher amounts in normal water conditions and yellow-seeded families had higher amounts under water stress conditions. Lignans are effective antioxidants, and they have the potential to scavenge harmful ROS that are usually overaccumulated under stress conditions [31,44]. Importantly, lignans could be used for medicinal purposes due to their antiviral properties, and their derivatives are also used in cancer chemotherapy [43]. The flax plant has incurred many costs to resist environmental stress, including producing more lignans. For example, family 71y had the highest seed yield under water stress conditions while the amount of SDG was increased in this family, which was observed in most of the studied families (Table 3 and Table 5).

### 3.3. Effect of Water Stress on the Association between Seed Quality Traits with Seed Coat Color

Biplot results showed a positive and significant relationship between seed yield and lignans under normal and water stress conditions, like with other secondary metabolites, making genotypes more resistant to environmental stress. The biplot under normal water conditions showed that families with higher mucilage and SDG had lower seed yields, seed oil content, and SECO. Additionally, as previously mentioned, water deficiency in plants leads to an increase in mucilage, fiber, protein, SECO, and SDG. Although seed yield decreased under water stress conditions, the nutritional value of the seeds increased, reflected by higher SDG, fiber, and protein in brown seeds and SECO, seed oil content, unsaturated fatty acids, and mucilage in yellow seeds. The biplot results also showed that the high amount of fiber, protein, and lignans in brown-seeded families made them more tolerant to stress than the yellow-seeded ones. The high amount of oil and unsaturated fatty acids in seeds may reduce plant tolerance to stress and make them more vulnerable. This was evident in the yellow-seeded families, consistent with other reports [45]. In general, the biplot under water stress conditions showed that families such as 26y and 34b were desirable for Muc, SECO, CEL, and ADF traits but had low potential in terms of SY, seed oil, HCEL, and SDG. In contrast, for higher seed yield under stress conditions, families 61b and 53b were more suitable. Under normal water conditions, families 26y and 83y had the highest values of seed yield, mucilage, oil, and SDG.

The heat mapping graphs were also consistent with the biplot diagrams. This showed that genotypes with high seed yield under water stress conditions had a high and positive relationship with the lignans, especially SDG. In contrast, seed yield had a negative relationship with the mucilage and seed oil content.

## 4. Materials and Methods

### 4.1. Plant Material, Experimental Site, Design, and Imposed Water Stress

The plant material used in this study consisted of brown and yellow seeds of 8 parental (Table 6), obtained from IPK gene bank (Germany), and 22 flax F4 families selected from segregating populations of a full 8 × 8 diallel cross between contrasting parental lines for seed and flower colors. The yellow- and brown-seeded progenies of each F_3_ line were planted adjacently in plots in which rows were two meters in length and 25 cm apart. All F_3_ lines along their parents were field planted according to a randomized complete block design with three replications under two moisture environments. The experimental site was the research farm of Isfahan University of Technology located 40 km southwest of Isfahan in Lavark, Najaf Abad region (latitude 32°32′ north and longitude 51°22′ east), 1630 m above sea level. For this site, the average annual temperature is 14 °C, and its annual rainfall is 140 mm, with a specific soil density of 1.34 g. cm^3^ and pH = 7.5. Data from the F3 lines were subjected to statistical analysis based on the studied traits, and F4 seeds from 22 families within three groups of the low, medium, and high seed yield were selected for this experiment.

The factor moisture environments (control 50% and drought stress 80%) were applied based on the maximum permissible drainage rate (MAD) of soil available water (SAW) [46]. Water stress treatment was applied from the late flowering stage until plant seed maturity (late April to the end of July). The irrigation intervals (days between two irrigations) were determined based on meteorological data and evapotranspiration records up to 60 cm deep in the soil.

Soil moisture was measured according to standard gravitational methods [46] at three depths of 0–20, 20–40, and 40–60 cm. The depth of irrigation was determined based on the following two equations:

θ_irri_ = θ _FC_- (θ _FC_ − θ _PWP_) × ρ × MAD (1)

where θ_irri_ is the mean soil moisture content at the root development depth at irrigation time under non-stress treatment, θ_FC_ is soil moisture content at field capacity, θ_pwp_ is soil moisture content at the wilting point, ρ =1.4 g cm^−3^, and MAD is the depletion of 50 or 80% of the total available water [47];

D_irrig =_ (θ_fc_ − θ_avg_) × D_rz_ (2)

in which D_irrig_ is the irrigation depth (cm), D_rz_ is the depth of the root zone (cm), and θ_avg_ is soil-water content at the root zone before irrigation (m^3^/m^3^). Irrigation was performed using a drip system, and the volume of water used in each treatment was measured using a volumetric counter.

### 4.2. HPLC-DAD Analysis of Lignans

Lignans are macromolecular compounds that must be hydrolyzed in the extraction pathway. High-performance liquid chromatography (HPLC) was used to evaluate the content of secoisolariciresinol diglucoside (SDG) and secoisolariciresinol (SECO) in flaxseed. The method described by Mukker et al. [48], with some modifications, was followed to prepare the samples for injection into the HPLC device (Agilent 1090, with a diode array detection (DAD) system, Santa Clara, CA, USA). For extraction of lignans, 250 mg of flaxseed sample was added to 2.5 mL of the extraction solvent (EtOH 75%, HPLC grade, Merck, Branchburg, NJ, USA) and mixed. The samples were then placed on ultrasound (45 kHz, 50 °C) for 1 h. The extracts were then allowed to cool down at room temperature for at least 30 min (or overnight in a cold room) and then neutralized (up to pH:7) using acetic acid. The extracts were centrifuged at 5000× *g* for 15 min, supernatant filtered through a 0.22 μm filter, and the extracts were prepared for injection in HPLC. The calibration curve was used to quantify the lignans, and the results were calculated as mg (secoisolariciresinol diglucoside) SDG per gram of dry weight. The coefficient of determination of the standard calibration curve was 0.999; limits of detection and quantification, as well as the validation of the method, were described by Anjum et al. [49]. Processed samples (50 μL) were injected into a Waters Symmetry C18 column (4.6 mm × 150 mm, 5 μm). The analytes were eluted under gradient mode with a mobile phase consisting of water with 0.1% formic acid (component A) and acetonitrile with 0.1% formic acid (component B) in different ratios delivered at a flow rate of 1 mL/min. The excitation wavelength was set at 278 nm. The mobile phase was degassed in an ultrasonic bath for 30 min before use. The column was maintained at room temperature (22 °C) and washed with water: methanol (50:50) after every use. The potential for autosampler carryover was reduced by the injection of a blank mobile phase after the highest calibration curve concentration. For SDG and SECO, the mobile phase gradient conditions consisted of an initial isocratic condition of 85:15 component A: component B from 0 to 12 min, an increasing gradient from 15% to 50% of component B from 12 to 14 min, and then 50–90% from 14 to 16 min, a decreasing gradient from 90% to 15% component B from 16 to 23 min, and a return to 85:15 component A: component B between 23 and 25 min [48]. To compare results with the published literature, glycosides such as SDG and SECO were expressed as mg equivalent per gram of dry weight (DW).

(3) $$\mathrm{Lignans}\;\mathrm{productivity}\;\frac{mg}{l}=DW(\frac{g}{l})\;\times \;\mathrm{accumulation}\;(\frac{mg}{g})$$

### 4.3. Oil Extraction, Protein, Fatty Acids, and Amino Acids Profiles

Oil was extracted from the ground flaxseeds using the Soxhlet extractor apparatus described in the AOAC (1990) [50] with n-hexane (60–80 °C) as a solvent.

Then, seed quality traits including fatty acid composition (palmitic acid (PA), oleic acid (OA), linoleic acid (LA) and α-Linolenic acid (ALA), protein content (PRO), fiber content (FIB), amino acid profile (aspartic acid (AA), methionine (Met), histidine (His), and arginine (Arg)), ash percentage, and moisture content were determined by near-infrared (NIR) spectroscopy analyzer with three replications. About 10–15 g of cleaned and intact seed from each of the 30 F4 families was placed in a small ring cup and scanned with NIR Systems model DA 7250-monochromator instrument (Perten Instruments, Hagersten, Sweden).

### 4.4. Mucilage Extraction of Flaxseed

Extraction of mucilage from flaxseed was performed using the method of Cui et al. [51], which was modified by Nasrabadi et al. [52].

Initially, 50 g of flaxseed was added to distilled water at 80 °C in a ratio of 1 to 13 (seed/water) and stirred for 2 h in a shaker incubator at 80 °C. The mucilage solution was then separated from the swollen grains using a sieve (mesh 40). To precipitate the mucilage, 96% ethanol was added to the mucilage solution in a ratio of 1:3 (mucilage solution/ethanol). The resulting precipitate was separated using a sieve (mesh 60). After solvent removal, the extracted mucilage was dried in a freeze dryer for 24 h at −40 °C, and the amount of seed mucilage was calculated using the ratio formulas [52].

### 4.5. Dietary Fiber Analysis in Flaxseed

For flaxseed samples, neutral detergent fibers (NDF), acid detergent fibers (ADF), and lignin (ADL) were determined for the following content of dietary fiber components. The NDF content was evaluated using a solution of a neutral detergent (sodium lauryl sulfate, ethylenediamine tetra acetic disodium salt, sodium borate, di-basic sodium phosphate, and triethylene glycol), alpha-amylase (17,400 liquid units/mL), and sodium sulfite [53]. The ADF content was determined using an acid detergent, cetyltrimethylammonium bromide (CTAB), and standardized sulfuric (VI) acid [54]. After determining the ADF content, the lignin content (ADL) was assayed by using a standardized solution of sulfuric (VI) acid [50]. The following formula was used to calculate the amount of each type of fiber:

(4) $$\%\;\mathrm{ADF}\;\mathrm{and}\;\%\;\mathrm{NDF}\;(\mathrm{as}-\mathrm{received}\;\mathrm{basis})=\left(\frac{W_{3}-\left(W_{1}\times C_{1}\right)}{W_{2}}\right)\times 100$$

In this formula, $W_{3}$ is bag weight and residue, $W_{2}$ is sample weight, $W_{1}$ is empty bag weight, and $C_{1}$ is the weight of the control bag.

The difference between the contents of NDF and ADF fractions was used to compute the hemicellulose (HCEL) concentration, and the difference between the contents of ADF and lignin (ADL) served to calculate the cellulose (CEL) concentration in flaxseed samples.

### 4.6. Statistical Analysis

First, the normality of the data was checked, and all the data were subjected to a combined analysis of variance using SAS statistical software (ver. 9.4; SAS Institute Inc. Cary, NC, USA) according to the design of the experiment. Secondly, GraphPad Prism (ver. 9.3.1) software was used to compare all genotypes’ mean and investigate the difference between genotypes based on seed color and moisture environment. The significant differences among the means were determined using the least significant difference (LSD) test (*p* < 0.05). Principal component analysis (PCA) was performed, and biplot and heat-mapping drawings were prepared to identify the interrelationships among the genotypes and measured traits using the Stat Graphics centurion XVIII (http://www.statgraphics.com, accessed on 1 February 2023). For grouping the genotypes and traits, JMP (ver.16) software was used.

## 5. Conclusions

Water stress and seed coat colors affected most of the studied traits of flax genotypes. Brown-seeded families showed more tolerance to water stress due to higher content of fiber, protein, and secondary metabolites such as lignans (SDG). Under normal and water stress conditions, the quantity and quality of the seed oil in yellow-seeded families were higher than in brown ones. However, in both seed-colored families, the amount of lignans increased due to moisture stress, particularly in brown seeds. Additionally, the amount of mucilage and seed fiber was inversely related to moisture stress and increased, and the increase in the amount of fiber was higher in brown seed ones. Under normal moisture conditions, yellow-seeded families had higher seed yield, oil yield, and general quality traits. Our findings suggest that yellow or brown seed varieties of flax may be selected for cultivation depending on the component and final product needed. Therefore, families 61b and 53b are suggested for higher seed yield, high lignans, and fiber and families 26y and 45y for higher seed oil, fatty acids, mucilage, and SECO.

## Figures and Tables

**Table 1:** Combined analysis of variance (ANOVA) for biochemical characteristics of flaxseeds over two environments.

S.O.V	df	Mean Squares									
		SY	Oil	NDF	ADF	ADL	CEL	HCEL	Muc	SDG	SECO
Moisture regimes (Environment)	1	169,195.4 **	333.53 **	41.79 **	0.02 ^ns^	52.86 **	54.91 **	40.1 **	235.01 **	113.9 **	6.92 **
Block (Environment)	2	126.43 ^ns^	12.62 ^ns^	13.77 ^ns^	4.08 ^ns^	0.39 ^ns^	5.14 ^ns^	14.01 ^ns^	0.024 ^ns^	0.26 ^ns^	0.05 ^ns^
Entries	29	3425.21 **	22.07 **	28.8 **	14.05 **	9.85 **	12.94 **	26.08 **	4.91 **	37.6 **^#27^	5.32 **^#27^
Family	21	3867.41 **	17.9 **	32.21 **	11.55 **	10.81 **	11.19 **	17.89 **	5.35 **	43.31 **^#19^	5.88 **^#19^
Yellow	9	4192.97 **	13.17 **	31.72 **	8.35 **	9.06 **	7.13 **	18.31 ^ns^	4.44 **	44.04 **^#9^	6.91 **^#9^
Brown	11	2224.41 **	22.52 **	34.16 **	15.18 **	12.26 **	14.25 **	17.45 **	6.46 **	45.45 **^#9^	4.01 **^#9^
Yellow vs. Brown	1	2701.64 **	9.72 **	14.59 **	0.27 **	10.46 ^ns^	14.13 ^ns^	18.89 ^ns^	11.3 **	66.92 **	4.53 **
Parent	7	479.23 ^ns^	34.39 **	22.67 **	22.7 **	6.08 **	12.49 **	53.34 *	0.53 **	27.1 **	1.67 **
Yellow	3	257.01 ^ns^	15.12 **	38.8 **	22.21 **	6.18 **	21.02 **	69.25 *	0.92 **	49.98 **	3.46 **
Brown	3	809.73 ^ns^	65.03 **	12.78 **	10.9 **	1.41 **	4.56 **	44.37 *	0.27 **	10.77 **	0.44 *
Yellow vs. Brown ^P^	1	154.39 ^ns^	0.3 ^ns^	3.99 ^ns^	59.54 **	19.79 ^ns^	10.67 ^ns^	32.57 ^ns^	0.18 ^ns^	7.77 **	0.021 ^ns^
Family vs. Parent	1	2089.29 **	24.19 *	7.33 *	4.51 ^ns^	10.01 **	8.08 **	17.20 **	5.49 **	23.48 **	11.42 **
Environment × Entries	29	1872.91 **	6.8 ^ns^	33.49 **	9.96 **	12.25 **	8.37 **	35.27 **	2.60 ^ns^	13.6 **^#27^	2.15 **^#27^
Environment × Yellow ^F^	9	4608.08 **	4.72 ^ns^	13.26 **	3.45 ^ns^	11.71 **	8.83 **	15.00 **	1.14 **	12.39 **^#9^	1.34 **^#9^
Environment × Brown ^F^	11	1474.36 **	4.61 ^ns^	40.23 **	11.66 **	16.09 **	6.18 **	45.16 **	3.89 **	3.73 **^#9^	0.69 **^#9^
Yellow vs. Brown × Environment ^F^	1	10,290.85 *	9.45 ^ns^	14.59 ^ns^	0.27 ^ns^	10.47 **	14.13 *	18.88 *	1.30 ^ns^	66.92 **	0.53 ^ns^
Environment × Yellow ^P^	3	511.09 ^ns^	6.45 ^ns^	55.93 **	11.93 **	6.81 **	7.88 **	61.57 **	1.47 **	1.51 *	1.95 **
Environment × Brown ^P^	3	2197.74 **	1.17 ^ns^	35.16 **	2.79 ^ns^	8.55 **	2.55 **	21.77 **	1.29 **	4.26 **	0.68 **
Yellow vs. Brown × Environment ^P^	1	1540.39 *	0.30 ^ns^	3.99 ^ns^	59.54 **	19.79 **	10.44 **	32.54 *	0.18 ^ns^	7.77 **	0.002 ^ns^
Error	58	742.59	4.4	4.68	2.26	1.03	1.97	4.52	0.69	0.49^#54^	0.15^#54^
CV (%)		13.21	7.33	6.81	9.7	12.91	15.53	12.97	7.66	4.3	3.66

^F^: Family genotype, ^P^: Parent genotype, ^#:^ df SDG and SECO; ^ns^ non-significant; * Significant at 0.05 level of probability; ** Significant at 0.01 level of probability; S.O.V: source of variance; df: degrees of freedom. Oil: oil content. NDF: neutral detergent fibers. ADF: acid detergent fibers. ADL: lignin. CEL: cellulose. HCEL: hemicellulose. Muc: mucilage. SY: seed yield. The main degrees of freedom sources are on the right side of the df column, the sub sources are in the center, and the components of the sub sources are on the left side of the df column.

**Table 2:** Combined analysis of variance (ANOVA) for biochemical characters (NIR) of flaxseed over two environments.

S.O.V	df	Mean Squares											
		OA	LA	ALA	PA	AA	FIB	PRO	Met	His	Arg	Moi	ash
Moisture regimes(Environment)	1	538.26 **	1590.46 **	3.22 ^ns^	1854.8 **	0.16 ^ns^	338.8 **	15,555.1 **	223.36 **	6.32 **	3.05 **	10.9 **	85.57 *
Block (Environment)	2	29.97 ^ns^	2.29 ^ns^	2.13 ^ns^	2.66 ^ns^	0.14 ^ns^	8.83 ^ns^	819.61 ^ns^	1.66 ^ns^	0.42 ^ns^	0.08 ^ns^	0.32 ^ns^	0.85 ^ns^
Entries	29	126.83 **	168.5 **	30.91 **	68.66 **	1.14 **	20.64 **	3307.50 **	1.48 *	1.33 **	0.41 **	0.55 *	14.32 **
Family	21	121.1 **	128.34 **	32.93 **	77.49 **	0.92 ^ns^	76.93 **	3158.20 **	1.58 **	1.5 **	0.81 **	0.5 ^ns^	15.73 **
Yellow	9	127.1 **	143.62 **	25.01 **	101.68 **	1.84 **	18.03 ^ns^	4315.02 **	1.09 *	1.35 **	0.52 **	0.79 **	14.19 **
Brown	11	106.67 **	127.2 **	38.82 **	57.75 **	1.82 **	87.55 **	2461.31 **	1.54 **	1.76 **	0.79 **	0.32 *	17.98 **
Yellow vs. Brown	1	167.94 **	142.15 **	58.22 **	33.95 ^ns^	0.79 ^ns^	36.78 **	997.75 ^ns^	12.39 **	0.96 *	0. 01 ^ns^	0.05 ^ns^	0.08 *
Parent	7	152.6 **	258.41 **	29.64 **	32.82 **	1.77 *	31.32 ^ns^	3055.79 **	2.83 **	0.79 **	0.14 **	0.29 ^ns^	6.22 ^ns^
Yellow	3	113.25 **	329.48 **	38.86 **	33.63 **	1.91 *	15.93 ^ns^	4246.02 **	1.89 *	0.45 **	0.19 *	0.42 ^ns^	12.68 *
Brown	3	235.86 **	259.48 **	81.26 **	42.82 **	0.74 ^ns^	77.07 ^ns^	2882.00 *	0.15 ^ns^	1.26 **	0.19 *	0.23 ^ns^	1.22 ^ns^
Yellow vs. Brown ^P^	1	40.65 ^ns^	0.31 ^ns^	66.15 **	0.002 ^ns^	3.68 *	21.04 ^ns^	5.45 ^ns^	0.6 ^ns^	0.42 ^ns^	2.28 **	0.002 ^ns^	12.8 ^ns^
Family vs. Parent	1	147.34 **	156.38 **	0.29 ^ns^	11.39 ^ns^	0.57 ^ns^	26.28 **	239.34 ^ns^	1.74 ^ns^	1.72 **	0.62 **	0.98 **	5.89 ^ns^
Environment × Entries	29	115.7 **	61.85 **	32.96 **	47.23 **	1.06 **	14.09 **	4424.01 **	1.43 *	0.95 **	0.44 **	0.28 ^ns^	13.58 **
Environment × Yellow ^F^	9	130.02 **	1324.5 **	25.98 **	771.49 **	2.01 **	47.43 ^ns^	27,277.6 **	80.31 **	4.63 **	0.73 **	5.55 **	15.64 **
Environment × Brown ^F^	11	31.85 ^ns^	276.32 **	22.59 **	662.38 **	0.03 ^ns^	99.27 **	48,694.02 **	94.61 **	0.8 **	0.55 **	4.49 **	34.05 **
Yellow vs. Brown × Environment ^F^	1	226.50 *	3.34 ^ns^	30.39 *	79.98 *	0.54 ^ns^	0.79 ^ns^	411.05 ^ns^	6.45 *	0.06 ^ns^	3.38 **	0.01 ^ns^	4.94 ^ns^
Environment × Yellow ^P^	3	548.49 **	158.04 **	0.64 ^ns^	163.58 **	2.13 *	286.9 **	52,675.01 **	17.09 **	0.07 ^ns^	3.33 **	0.84 *	15.81 *
Environment × Brown ^P^	3	151.59 **	106.34 **	34.72 **	227.5 **	6.83 **	28.48 ^ns^	42,690.8 **	33.37 **	2.83 **	0.99 **	0.52 ^ns^	56.73 **
Yellow vs. Brown × Environment ^P^	1	21.04 ^ns^	40.65 ^ns^	66.15 **	0.30 ^ns^	3.68 **	0.20 ^ns^	5.54 ^ns^	0.60 ^ns^	0.43 *	0.09 ^ns^	0.002 ^ns^	1.80 ^ns^
Error	58	21.44	16.22	7.20	0.29	0.74	34.57	773.91	0.74	0.16	0.05	0.29	4.57
CV (%)		6.07	6.08	8.36	5.61	5.25	9.12	9.1	5.25	8.5	6.1	5.61	8.93

^F:^ Family genotype, ^P:^ Parent genotype; ^ns^ non-significant; ^*^ Significant at 0.05 level of probability; ^**^ Significant at 0.01 level of probability; S.O.V: source of variance; df: degrees of freedom. PRO: protein content. FIB: fiber content. OA: oleic acid. LA: linoleic acid. PA: palmitic acid. ALA: α- Linolenic acid. Met: methionine. His: histidine. Arg: arginine. AA: aspartic acid. Moi: moisture. The main degrees of freedom sources are on the right side of the df column, the sub sources are in the center, and the components of the sub sources are on the left side of the df column.

**Table 3:** Means of quality traits in parents and families of flax under control and intense drought stress (IDS).

Parent/ Family	SY(g/m2)		Oil (%)		ADF (%)		ADL (%)		CEL (%)		HCEL (%)		Muc (g/100 g)		NDF (%)	
	N	S	N	S	N	S	N	S	N	S	N	S	N	S	N	S
B1	172.98	106.12	**41.7 ^max^**	32.41	12.21	13.12	5.21	7.83	7.22	7.66	19.62	18.83	9.71	10.25	31.83	34.33
B2	165.81	108.03	**25.7 ^min^**	26.02	14.35	14.53	7.07	6.43	7.35	8.62	18.8	15.16	9.75	11.75	31.17	31.5
B3	191.21	162.08	32.32	34.63	15.12	18.27	4.66	9.88	10.33	8.39	12.12	12.56	11.5	12.71	27.17	30.83
B4	198.76	120.11	33.81	28.51	12.23	16.17	4.52	8.65	7.73	6.52	**24.4 ^max^**	14.52	10.25	12.51	30.67	34.65
Y5	157.81	130.75	26.11	31.22	16.17	16.66	7.66	8.65	7.01	8.51	16.17	10.8	11.11	11.62	32.33	35.17
Y6	172.53	140.02	34.4	**34.9 ^max^**	14.83	14.99	8.33	6.17	6.51	8.22	12.17	**24.9 ^max^**	12.25	12.51	27.19	**39.3 ^max^**
Y7	253.75	131.65	33.81	33.3	17.67	**23.3 ^max^**	7.67	7.21	10.1	**12.3 ^max^**	10.17	8.33	10.12	12.85	25.83	31.65
Y8	174.25	108.52	34.33	32.81	14.83	21.12	7.66	9.17	7.17	9.83	18.51	17.66	10.75	12.75	33.35	38.62
12b	201.32	147.75	32.22	33.23	**19.6 ^max^**	21.67	9.56	7.33	11.22	10.33	11.52	22.52	9.9	13.11	31.17	38.17
13b	190.48	170.31	31.35	33.53	17.05	18.76	7.66	7.17	9.39	5.33	**8.61 ^min^**	14.33	6.55	12.58	**25.6 ^min^**	26.67
17y	268.22	145.42	37.21	32.78	12.55	13.35	5.83	6.02	5.72	7.35	16.84	18.15	8.62	11.61	29.42	32.5
23b	162.58	123.35	32.56	25.42	16.83	17.16	7.53	10.6	9.35	3.54	12.65	24.16	7.04	12.02	29.53	38.73
26y	**130.1 ^min^**	**34.23 ^min^**	32.46	**25.1 ^min^**	18.15	18.51	10.66	9.13	7.48	9.51	17.18	14.51	**12.7 ^max^**	**13.7 ^max^**	32.33	34.02
28b	197.66	121.17	26.46	23.11	**11.8 ^min^**	**10.3 ^min^**	7.67	**5.1 ^min^**	8.16	5.16	19.35	16.51	9.15	12.25	35.16	36.83
32b	171.81	114.22	27.12	26.12	15.15	16.52	**11.1 ^max^**	10.3	**5.63 ^min^**	5.66	20.16	19.16	8.57	12.5	35.67	33.65
34b	191.82	100.31	35.14	26.62	15.17	22.17	7.56	**17.3 ^max^**	8.22	4.84	18.65	16.76	11.25	13.25	34.71	38.33
43b	206.25	139.27	33.72	27.34	17.21	19.47	10.25	7.33	6.75	9.66	21.15	13.67	9.25	12.25	**38.5 ^max^**	34.65
45y	147.11	93.11	33.87	27.08	14.16	16.33	8.16	9.42	6.06	7.33	16.45	13.33	11.63	13.25	28.83	30.67
47y	189.42	149.27	32.76	29.12	12.94	11.52	6.16	7.33	4.78	6.16	18.18	13.33	8.75	12.52	31.83	24.83
47b	217.71	199.14	36.65	28.91	14.5	15.83	7.16	10.9	7.35	4.89	19.16	15.16	7.25	**8.75 ^min^**	33.67	34.11
48b	175.83	171.56	33.65	**33.71**	16.52	16.33	8.52	9.93	8.21	8.52	14.83	19.13	10.23	11.53	31.35	35.49
53b	188.44	169.21	32.96	27.41	15.35	17.83	7.66	8.17	7.66	6.67	16.35	20.35	**6.06 ^min^**	11.01	31.65	36.52
56b	155.11	147.43	31.22	25.24	16.66	18.38	9.44	8.83	7.65	5.33	15.12	15.21	11.56	**13.7** **^max^**	31.66	33.77
61b	251.47	**197.7 ^max^**	35.31	31.34	14.98	16.78	7.12	11.95	7.98	3.72	22.02	13.33	8.36	11.52	37.12	39.12
71y	234.72	95.36	**39.92**	33.13	14.33	15.03	6.65	7.16	7.66	6.83	14.83	12.65	8.51	12.5	29.16	27.66
72y	238.22	110.22	32.64	25.67	15.06	14.83	**3.3 ^min^**	8.5	**12.6 ^max^**	4.16	17.11	13.15	8.54	12.25	30.17	29.83
75y	252.02	105.14	34.33	26.12	13.65	17.67	5.33	11.33	8.33	**3.33 ^min^**	19.16	**8.83 ^min^**	8.75	12.5	32.83	26.54
81y	161.68	152.2	36.12	33.63	17.17	16.56	7.17	7.34	10.12	9.16	19.83	21.65	8.5	12.51	37.13	38.16
82y	220.71	143.51	33.14	25.25	15.19	14.66	3.83	6.85	8.16	7.81	11.16	11.83	8.54	12.75	36.36	32.51
83y	**271.1 ^max^**	137.02	33.62	26.11	15.88	16.83	7.21	7.21	5.64	6.83	15.78	11.83	8.36	12.11	31.66	**24.6 ^min^**
LSD (%)	31.72	28.86	3.28	2.19	1.89	1.68	0.52	1.55	0.91	1.77	4.08	4.5	0.95	1.06	1.19	1.09

Oil: oil content. NDF: neutral detergent fibers. ADF: acid detergent fibers. ADL: lignin. CEL: cellulose. HCEL: hemicellulose. Muc: mucilage. SY: seed yield. S: stress condition. N: normal condition. ^max^: The maximum mean value of the trait. ^min^: The minimum mean value of the trait.

**Table 4:** Means of quality traits (NIR) in parents and families of flax under control and intense drought stress (IDS).

Parent/ Family	OA (ω-9) (%)		LA (ω-6) (%)		ALA (ω-3) (%)		Rate(ω-6)/(ω-3)		PA (%)		FIB (%)		Ash (%)	
	N	S	N	S	N	S	N	S	N	S	N	S	N	S
B1	13.36	14.67	26.76	31.96	27.71	30.86	0.97:1	01:01	7.09	6.88	13.36	14.57	10.12	16.29
B2	14.32	15.53	21.87	30.33	38.14	27.48	0.7:1	01:01	6.46	8.06	11.39	16.69	11.87	13.97
B3	17.01	16.00	22.08	31.50	32.18	30.16	0.73:1	0.97:1	4.96	6.01	9.71	13.77	13.31	15.67
B4	**8.2 ^min^**	11.31	44.5	42.05	37.47	32.39	**1.9:1 ^max^**	1.2:1	8.06	7.95	17.02	22.14	10.03	18.3
Y5	18.13	15.18	38.66	**55.3 ^max^**	28.73	36.12	1.3:1	1.7:1	5.46	6.4	8.91	**23.01 ^max^**	14.52	17.33
Y6	15.14	18.18	28.17	**28.1 ^min^**	32.50	37.27	0.66:1	0.92:1	4.99	5.93	12.60	16.54	9.18	17.11
Y7	12.52	10.47	**20.7 ^min^**	35.06	29.71	34.53	0.7:1	01:01	8.21	7.71	9.96	14.03	17.96	14.56
Y8	16.47	11.21	34.47	28.67	28.36	31.48	1.2:1	**0.9:1^min^**	5.57	6.76	9.06	15.81	10.21	13.57
12b	13.17	16.51	36.68	31.51	34.54	31.98	01:01	0.98:1	6.59	7.78	**19.19 ^max^**	17.73	14.43	13.65
13b	19.55	11.36	34.56	40.25	36.87	**25.2 ^min^**	01:01	1.6:1	7.47	7.47	9.63	15.53	17.81	16.95
17y	19.18	17.52	36.52	47.87	27.86	**37.6 ^max^**	1.3:1	1.3:1	5.21	**9.01 ^max^**	16.01	15.07	13.98	17.15
23b	15.34	13.07	21.06	45.14	35.22	30.60	0.65:1	1.7:1	7.09	6.56	11.90	**22.04**	**19.45 ^max^**	16.66
26y	19.35	**9.4 ^min^**	30.12	41.02	33.08	36.46	0.9:1	1.5:1	6.46	7.67	13.13	14.55	17.18	**10.67 ^min^**
28b	18.28	11.90	24.31	36.45	32.07	32.04	0.76:1	01:01	5.56	8.56	12.32	17.06	11.96	13.90
32b	10.11	18.78	33.78	33.52	35.46	31.51	1.1:1	01:01	4.02	7.67	13.12	16.57	18.68	15.40
34b	14.44	10.39	42.38	42.52	37.87	27.13	1.5:1	0.97:1	8.17	5.19	9.96	15.29	14.46	17.18
43b	**20.6 ^max^**	11.74	**49.09 ^max^**	54.28	30.11	31.53	1.6:1	1.6:1	7.57	7.21	9.02	16.46	9.25	17.58
45y	16.10	12.91	32.07	45.08	33.01	35.37	0.97:1	1.7:1	6.82	4.47	10.02	13.25	**9.01 ^min^**	16.98
47y	19.74	**22.7 ^max^**	30.95	37.22	29.60	35.42	01:01	1.5:1	7.06	5.79	**8.17 ^min^**	14.08	17.74	17.34
47b	12.79	13.07	34.58	33.37	35.24	31.76	0.98:1	01:01	6.12	4.18	12.41	15.02	18.55	19.81
48b	14.07	14.89	35.61	41.91	37.32	27.63	01:01	01:01	5.71	**3.02 ^min^**	9.71	16.11	12.13	15.9
53b	11.30	16.51	27.77	41.69	25.85	33.01	01:01	1.5:1	6.04	5.46	10.36	16.37	9.02	17.05
56b	12.15	19.81	37.90	31.48	**44.42 ^max^**	35.55	0.85:1	1.3:1	8.47	5.13	14.04	15.89	16.78	16.3
61b	14.87	18.22	33.31	36.53	34.07	30.81	01:01	1.2:1	6.89	8.63	9.41	**10.53 ^min^**	9.22	15.51
71y	16.80	17.87	28.14	33.32	25.27	31.3	1.1:1	01:01	7.70	6.52	13.32	11.85	16.41	16.20
72y	14.02	17.13	22.49	32.35	35.03	32.02	**0.6:1 ^min^**	01:01	5.12	4.46	14.46	17.27	19.04	15.56
75y	13.68	20.82	41.73	48.12	33.99	36.05	1.2:1	**1.8:1 ^max^**	6.46	5.46	11.96	10.86	11.12	16.96
81y	17.57	14.89	30.51	48.45	32.85	34.53	0.9:1	1.5:1	**3.7 ^min^**	6.86	14.71	17.98	17.92	**19.91 ^max^**
82y	17.81	10.23	22.36	34.73	24.05	27.68	0.9:1	1.2:1	4.15	4.07	10.84	11.57	17.56	17.60
83y	11.52	20.27	30.21	52.03	**22.32 ^min^**	32.97	1.3:1	1.6:1	**9.6 ^max^**	7.04	10.31	15.23	14.01	14.96
LSD (%)	3.66	2.03	5.04	6.95	5.81	3.14	-	-	5.96	2.01	2.27	2.64	2.36	4.37

OA: oleic acid. LA: linoleic acid. ALA: α- Linolenic acid. PA: palmitic acid. FIB: fiber content. S: stress condition. N: normal condition. ^max^: The maximum mean value of the trait. ^min^: The minimum mean value of the trait.

**Table 5:** Means of quality traits (NIR) and lignans in parents and families of flax under control and intense drought stress (IDS).

Parent/ Family	PRO (mg. g^−1^)		AA (%)		Met (%)		His (%)		Arg (%)		Moi (%2)		SDG (mg/g)		SECO (mg/g)	
	N	S	N	S	N	S	N	S	N	S	N	S	N	S	N	S
B1	129.54	254.48	1.9	**5.01 ^max^**	1.93	4.09	1.77	3.33	0.58	1.83	3.01	3.69	11.29	15.63	4.8	3.22
B2	135.96	213.95	2.84	2.73	1.18	4.43	1.67	2.53	**0.28 ^min^**	1.82	3.12	3.77	11.17	13.26	5.12	4.33
B3	**104.9 ^min^**	189.06	2.48	3.66	1.55	4.41	**0.91 ^min^**	2.09	0.82	1.72	**3.81 ^max^**	3.55	13.82	16.16	5.07	3.31
B4	146.3	273.93	1.92	2.97	1.46	5.01	1.45	1.2	0.48	1.56	2.9	3.29	12.66	19.19	4.55	4.6
Y5	139.4	**307.5 ^max^**	2.27	1.49	3.03	4.49	2.61	1.33	0.9	0.6	3.46	3.5	12.57	17.62	5.12	5.4
Y6	120.03	168.49	**2.95 ^max^**	2.57	1.48	3.44	1.21	3.45	1.57	0.29	3.08	3.72	17.99	**22.21 ^max^**	5.48	4.54
Y7	112.95	254.93	**2.95 ^max^**	2.3	1.95	5.58	2.36	2.48	1.2	0.55	2.87	3	10.27	16.29	2.56	3.89
Y8	125.55	226.04	1.9	1.67	2.41	3.64	1.94	1.45	1.84	0.41	3.12	4.26	10.51	13.63	2.92	5.24
12b	139.66	257.91	1.93	2.83	**3.22 ^max^**	5.96	1.83	1.5	0.56	1.81	3.05	3.38	NE	NE	NE	NE
13b	138.57	211.53	2.76	2.48	2.52	**6.45 ^max^**	2.46	1.95	0.58	1.77	2.55	3.07	9.03	**9.51 ^min^**	2.43	1.14
17y	147.52	215.5	2.73	1.65	2	5.48	1.7	3.16	1.23	**1.85 ^max^**	2.63	3.94	9.94	12.25	1.78	2.19
23b	259.47	259.98	**1.23 ^min^**	2.34	1.53	6.04	1.62	**0.76 ^min^**	0.42	1.67	2.03	4.09	NE	NE	NE	NE
26y	136.51	199.88	2.81	2.5	2.16	3.31	1.4	1.44	1.56	0.82	2.75	**2.80 ^min^**	11.53	13.58	**1.67 ^min^**	4.52
28b	128.57	252.05	1.35	1.07	2.08	5.45	2.71	1.69	0.58	**0.21 ^min^**	2.98	3.15	NE	NE	NE	NE
32b	127.1	291.28	2.25	1.34	1.26	5.96	1.55	3.79	0.31	1.69	2.37	3.37	17.22	20.05	3.11	1.24
34b	147.99	245.38	**2.89**	2.5	2.34	3.85	2.6	2.96	0.45	1.82	2.53	3.73	14.24	18.21	2.98	1.55
43b	152.22	193.05	2.67	1	3.06	4.49	**3.57 ^max^**	3.45	0.9	1.78	4.02	3.58	14.5	17.22	4.67	2.48
45y	187.82	218.72	2.06	1.84	2.25	4.13	2.23	2.66	2.36	0.51	3.54	3.43	18.11	15.45	4.77	5.28
47y	**272.1 ^max^**	207.99	2.08	1.24	2.31	6.37	1.42	2.64	1.07	0.81	**1.08 ^min^**	2.85	13.06	11.88	4.75	3.44
47b	184.21	216.73	1.78	3.15	3	6.27	1.55	2.39	0.5	0.83	3.25	3.28	9.17	15.73	**5.63 ^max^**	3.41
48b	249.74	214.56	2	2.63	2.51	**2.96 ^min^**	2.2	3.48	1.51	1.62	3.08	3.77	10.6	14.06	1.57	**1.03 ^min^**
53b	258.56	**153.7 ^min^**	1.9	1.27	1.43	5.07	1.13	1.48	0.68	1.75	2.98	4.17	8.26	15.14	5.13	2.84
56b	129.77	222.95	2.47	2.87	2.52	4.03	1.96	2.12	0.34	1.83	3.14	3.46	18.89	21.56	4.69	2.47
61b	127.42	288.55	2.05	1.1	2.96	5.59	1.8	2.53	0.41	1.72	3.02	3.75	13.67	18.76	3.68	2.15
71y	120.32	172.48	2.07	2.6	1.2	4.98	2.08	2.87	0.64	0.83	2.9	3.91	**6.37 ^min^**	10	2.91	3.99
72y	133.03	246	2.19	1.93	**1.09 ^min^**	5.98	1.52	2.8	0.88	1.18	3.33	3.56	16.36	11.29	1.85	2.88
75y	161.02	293.46	2.54	2.53	1.55	3.71	2.95	2.15	0.45	1.07	2.96	3.9	8.95	11.61	4.81	5.49
81y	255.5	239.58	2.46	3.26	1.65	3.98	1.34	2.26	0.94	0.48	2.61	3.19	NE	NE	NE	NE
82y	141.28	202.12	1.91	**0.98 ^min^**	2.4	3.49	2.07	1.51	**2.56 ^max^**	0.84	2.91	3.88	NE	NE	NE	NE
83y	161.53	243.23	1.65	3.67	1.46	4.99	2.55	**4.54 ^max^**	0.57	1.15	3.18	**4.62 ^max^**	**19.15 ^max^**	15.94	4.81	**5.72 ^max^**
LSD (%)	15.434	37.82	0.53	0.95	0.73	1.35	0.85	0.78	0.45	0.43	0.95	1.02	1.36	1.51	0.87	0.74

PRO: protein content. AA: aspartic acid. Met: methionine. His: histidine. Arg: arginine. Moi: moisture. SDG: secoisolariciresinol diglucoside. SECO: Secoisolariciresinol. S: stress condition. N: normal condition. ^max^: The maximum mean value of the trait. ^min^: The minimum mean value of the trait.

**Table 6:** Parental information used in the 8 × 8 diallel cross with respect to seed and flower colors.

Parents	IPK Code	Origin	Flower Color	Seed Color
B1	Ind111	India	White	Brown
B2	LTU1474	Lithuania	White	Brown
B3	Ko37	Iran	Blue	Brown
B4	FRA806	France	Blue	Brown
Y5	FRA771	France	White	Yellow
Y6	USA1580	United States	White	Yellow
Y7	Sp1066	Canada	Blue	Yellow
Y8	Gol12	Canada	Blue	Yellow

B: brown seed, Y: yellow seed.

## Data Availability

All data supporting the findings of this study are available within the paper.
